# A pilot Randomized Controlled Trial (RCT) study protocol for assessing physical activity in individuals diagnosed with Borderline Personality Disorder (PABORD)

**DOI:** 10.1192/j.eurpsy.2024.372

**Published:** 2024-08-27

**Authors:** A. Martinelli, M. D’Addazio, S. Leone, R. Rossi, S. Pogliaghi, G. Marchitelli, M. Zamparini, G. Moncalieri, E. Toffol, G. de Girolamo

**Affiliations:** ^1^IRCCS Istituto Centro San Giovanni di Dio Fatebenefratelli, Brescia; ^2^University of Verona, Verona; ^3^ASST Pavia, Pavia, Italy

## Abstract

**Introduction:**

Most treatments for severe mental disorders involve either pharmacotherapy or psychological interventions, which show mild to moderate effectiveness and may not lead to complete remission. Physical activity (PA), effective in enhancing physical health among the general population, emerges as a potential adjunctive treatment option that can address the existing gaps.

Borderline Personality Disorder (BPD) is a severe condition associated with profound psychosocial impairment, a heightened risk of suicide, and considerable burden on informal caregivers and mental health service providers. While there is a lack of approved medications for individuals with BPD, psychosocial interventions demonstrated good efficacy. However, the implementation of these treatments is limited by the demanded extensive training for staff. No studies have investigated the effectiveness of structured PA as an adjunctive treatment for individuals with BPD.

**Objectives:**

The primary objective of this study is to assess whether the intervention group outperforms the control group in terms of improvement on a standardized assessment scale evaluating BPD psychopathology, the *Zanarini Rating Scale for Borderline Disorder.* Secondary objective is to assess whether the intervention group can increase and sustain higher levels of PA. We hypothesise that a structured PA program will demonstrate superior results compared to the psychoeducation control group concerning PA levels upon completion of the intervention. Additionally, we hypothesise that the intervention group will exhibit enhanced outcomes in psychopathology, functioning, and sleep.

**Methods:**

The PABORD Randomized Controlled Trial is designed for female outpatient individuals diagnosed with BPD aged 18-40 years. This trial will involve two distinct groups: (i) an intervention group (25 participants) that will engage in a 12-week structured PA program under the supervision of a sports medicine physician; (ii) a control group (25 individuals) that will undergo a 12-week psychoeducation program focused on PA and diet.

Patients are assessed at three different time points. Standardized assessments include psychopathology, psychosocial functioning, sleep, menstrual cycle and nutrition data. Measurements are taken on the amount and intensity of PA and sleep patterns using a biosensor device (Actigraph GT9X), dynamometric measures and BMI. Biomarkers and hormonal cycles are examined through the collection of plasma and saliva samples.

The trial is financially supported through donations (5x1000 fund), and has been submitted to the local Ethics Committee for approval. The trial registration process is also currently in progress.

**Results:**

Not yet available.

**Conclusions:**

The study will provide new knowledge which may enhance our treatment options with patients suffering from BPD.

**Disclosure of Interest:**

None Declared

